# Oxidative Quality of Acid Oils and Fatty Acid Distillates Used in Animal Feeding

**DOI:** 10.3390/ani11092559

**Published:** 2021-08-31

**Authors:** Elisa Varona, Alba Tres, Magdalena Rafecas, Stefania Vichi, Roser Sala, Francesc Guardiola

**Affiliations:** 1 Nutrition, Food Science and Gastronomy Department-XIA, Campus de l’Alimentació Torribera, Facultat de Farmàcia i Ciències de l’Alimentació, Universitat de Barcelona, Av Prat de la Riba, 171, 08921 Santa Coloma de Gramenet, Spain; evarona@ub.edu (E.V.); stefaniavichi@ub.edu (S.V.); fguardiola@ub.edu (F.G.); 2 Institut de Recerca en Nutrició i Seguretat Alimentària (INSA-UB), Universitat de Barcelona, Av Prat de la Riba, 171, 08921 Santa Coloma de Gramenet, Spain; magdarafecas@ub.edu; 3 Nutrition, Food Science and Gastronomy Department-XIA, Facultat de Farmàcia i Ciències de l’Alimentació, Universitat de Barcelona, Av Joan XXIII, 27-31, 08028 Barcelona, Spain; 4 Animal Nutrition and Welfare Service (SNiBA), Animal and Food Science Department, Facultat de Veterinària, Universitat Autònoma de Barcelona, Edifici V, Travessera dels Turons, 08193 Bellaterra, Spain; roser.sala@uab.cat

**Keywords:** fat byproducts, acid oils, fatty acid distillates, characterization, oxidative status, oxidative stability, feeds, animal nutrition

## Abstract

**Simple Summary:**

Feed producers and farmers seek alternative and economical fat ingredients to supply animals with energy and beneficial components, such as natural antioxidants. Some byproducts from the edible oil refining industry, such as acid oils (AO) and fatty acid distillates (FAD), fulfil these requirements, but differences in the animal performance have been reported as their main drawback, which might be due to their high variable composition, including their oxidation status. Therefore, the valorization of these byproducts as feed ingredients requires ensuring standardized products with adequate quality in terms of oxidation parameters. In this study, 92 AO and FAD were characterized, finding a huge variability in their oxidation status and stability. They all showed low primary oxidation values (peroxide values). The content of secondary oxidation compounds was higher in FAD (which are released from physical refining processes) than in AO (which originate from chemical refining), while polymeric compounds were higher in the latter. The fatty acid and tocol compositions that were related with the botanical origin influenced their oxidative stability. Thus, in the quality control of these products, apart from the compositional parameters, it is recommended to include the evaluation of the oxidation status, both by primary and secondary oxidation parameters.

**Abstract:**

Acid oils (AO) and fatty acid distillates (FAD) are byproducts from chemical and physical refining of edible oils and fats, respectively. Their high energy value makes their upcycling interesting as alternatives to conventional fats in animal feeding. The objective of this study is to characterize their oxidative quality and to provide recommendations about their evaluation for animal feeding purposes. The oxidation status (peroxide value (PV), *p*-Anisidine value (*p*-AnV), % polymeric compounds (POL)), the oxidative stability (induction time by the Rancimat at 120 °C (IT)), the fatty acid composition (FA), and tocopherol and tocotrienol content of 92 AO and FAD samples from the Spanish market were analyzed. Both AO and FAD showed low PV (0.8 and 1 meq O_2_/kg); however, *p*-AnV was higher in FAD (36.4 vs. 16.4 in AO) and POL was higher in AO (2.5% vs. not detected in FAD) as a consequence of the type of refining process. The botanical origin of AO and FAD influenced FA and tocol composition, and they influenced IT. A high variability was observed for most analyzed parameters, reinforcing the need for standardizing AO and FAD to obtain reliable feed ingredients and to include primary and secondary oxidative parameters within their quality control.

## 1. Introduction

Fats are relevant feed ingredients because of their high energetic and nutritional values, as they supply vitamins and essential fatty acids (FA), such as linoleic (C18:2 n-6) and linolenic acids (C18:3 n-3) [1]. However, when lipid oxidation occurs in these fat ingredients, several deteriorative changes in their chemical composition, sensorial properties, and nutritional value also take place. Lipid oxidation depends on availability of substrates (oxygen and oxidable compounds, such as polyunsaturated fatty acids (PUFA)) and on the balance between antioxidants (such as tocopherols (T), among others) and pro-oxidants (such as certain mineral elements). Thus, the oxidation process is influenced by factors inherent to fats and oils (e.g., FA profile, free fatty acid (FFA) content, antioxidants, and pro-oxidants), and by external factors, such as processing and storage conditions (light exposure, temperature, and oxygen availability) [2,3].

When lipid oxidation occurs in a fat, it might be accompanied by a reduction in its nutritional value, related to the loss of PUFA and antioxidants, and to an increase in oxidation compounds, which implies a loss of energy value. Several detrimental biological effects have been described for some oxidation compounds formed in vivo or absorbed from the diet [4,5]. Kanner [6] reported that many of these lipid oxidation products may be absorbed by animals after the dietary intake of oxidized lipids, especially if they are secondary oxidation compounds rather than hydroperoxides, as the latter in the stomach may decompose to alcohols and aldehydes, which will be further partly absorbed through the intestines. Moreover, digestibility impairments at high polymer contents have been reported, resulting from a lower efficiency of pancreatic lipase over them [7,8]. Feeding poultry with different oxidized oils has been described to induce metabolic oxidative stress and negatively affect growth performance [9]. Nevertheless, it seems that the effects on the animal performance vary depending on the degree of oil oxidation, the type of oxidation compounds, the length of the feeding period [10,11], the FA profile, the dietary fat inclusion rate, and the inherent presence of antioxidants/pro-oxidants, among others [12]. Moreover, diets containing oxidized oils might cause a reduction in the T content, which could affect meat T content, resulting in a decrease in its oxidative stability [13,14,15,16,17]. Nevertheless, according to Billek [18] feeding oxidized oils only led to toxic effects in animals when these oils were extremely overheated and had a very high content of oxidized compounds.

The presence of lipid oxidation products in feeds may be a consequence of their formation during feed manufacturing and storage, or it may come from some of the feed ingredients, such as the added fat. Crude oils are usually used as feed fats, but feed producers and farmers are interested in finding more economical and sustainable feed fats, being the co- and byproducts of the fat industry among the candidates [19]. For instance, acid oils (AO) and fatty acid distillates (FAD) are fat byproducts from the refining processes of edible fat and oils, and are included in the European Catalogue of feed materials [20]. Concretely, they are obtained from the steps meant to remove FFA from the crude oil: AO from the alkali neutralization step in chemical refining (after the acidification of the soap stocks), and FAD from the deodorization step in physical refining [21,22,23]. Soap stocks and FAD are released from the refining processes at different proportions depending on the level of FFA in the crude oils; in alkali neutralization, the soap stocks usually represent 1.6–2% of the crude oil, while the palm FAD (PFAD) represents 4–5% [24,25]. Thus, one of the ways to avoid them becoming waste products is to upcycle them, incrementing their use as high-energy ingredients for feeds. This is a more sustainable application than their use by the oleochemical industry to obtain different derivative products, since, for this latter purpose, a subsequent chemical and/or physical processing is always necessary. Thus, they could be an alternative to conventional fat and oils used in animal nutrition due to their lower prices and their suitable nutritional performance [26]. However, for this purpose, their quality, energy, and nutritional value should be guaranteed to assure competitive productive parameters and an adequate quality of the foods obtained. The main constituents of AO and FAD are FFA, which are higher in FAD than in AO [1,19]. They also contain variable amounts of neutral oil, moisture, impurities, and minor compounds, such as T, among others [1,27,28]. Despite high FFA contents being related to a reduction in FA digestibility and absorption, especially for SFA, and also, consequently, to a reduction in the dietary energy value of the fat sources [29,30], several studies have demonstrated the potential use of these FFA-rich byproducts (AO and FAD) without impacting FA utilization by animals, especially in adults, as long as the diets do not exceed a determined FFA content and unsaturated/saturated fatty acid (U/S) ratio [31,32] (for instance, in grower–finisher broilers, diets of up to 35% FFA using saturated or unsaturated fat sources [31], or up to 30% FFA and a U/S ratio of 2.61 using PFAD [32]). On the other hand, these byproducts show a non-standardized quality, because refining conditions are constantly being improved to increase yields and quality of refined oils and this, consequently, influences the composition and quality of the final byproducts [33,34]. Furthermore, the management of these byproducts in refineries seeks simplicity, and often, when several oils are refined, soap stocks or FAD are wasted in the same tank, which complicates the standardization of the final product [1]. Thus, the final composition of AO and FAD is highly variable, and feed producers and farmers are reluctant to use them as reliable feed ingredients.

To overcome this, a standardization of the AO and FAD present in the market would be necessary, including the evaluation of their oxidation status. However, despite the effects described above when feeding oxidized oils, feed fat regulations or guidelines usually do not include any lipid oxidation parameter [20,35], or only recommend the evaluation of the peroxide value (PV) [36], which is a primary oxidation parameter and, therefore, it is completely insufficient as a global measure of fat oxidation [19,37,38,39]. Moreover, AO and FAD quality is not always controlled or reported, nor are the sources of variability of their composition and oxidative stability known. In Varona et al. [1], AO and FAD from the Spanish market were collected and characterized to evaluate the parameters relevant for their energy value, finding some differences in their composition, such as for moisture and FFA content, that were mainly related to the refining process, while others (unsaponifiable matter, FA, and T composition) were more related to the botanical origin. Furthermore, all AO and FAD showed a high content of insoluble impurities [1]. Therefore, our hypothesis is that these differences in composition could lead to differences in the oxidative status and susceptibility to oxidation of these fats, and that the refining process and botanical origin of the crude oil might determine the type and amount of oxidation compounds accumulated in these byproducts. Thus, the main objective of this study is to assess the oxidation status and the oxidative stability of these byproducts to determine if they are influenced by the refining process and botanical origin of the oil, and to establish recommendations on the control of the oxidation parameters.

## 2. Materials and Methods

### 2.1. Samples

The same sample set used in Varona et al. [1] to evaluate the nutritional value of AO and FAD was used for this study. It included a total of 92 FFA-rich byproducts of edible oil refining intended for animal feeding and representative of the Spanish market. Of them, 79 samples were byproducts from chemical refining (AO) and 13 from physical refining (FAD). They were collected from edible oil refineries, AO producers (companies that buy AO and soap stocks to refineries and produce custom AO for different animal species), and feed producers. As a result of the usual availability of these products in the market, only 43 samples came from a single fat or oil, while most of the samples were blends obtained from the refining of different edible fats and oils (Table 1).

When the samples arrived at the laboratory, they were melted, homogenized, divided into smaller vials whose head space was filled with N_2,_ and stored at −20 °C until analysis. The melting conditions were adapted to the botanical origin of each fat according to Varona et al. [40], selecting the minimum temperature and heating time that guaranteed an optimal sample homogenization and minimized sample damage.

### 2.2. Methods

The analysis of PV, *p*-Anisidine value (*p*-AnV), polymeric compounds (POL), induction time measured by Rancimat at 120 °C (IT), FA composition and T and tocotrienol (T3) contents were conducted in duplicate in all AO and FAD samples. All detailed analytical protocols can be found in Varona et al. [40] and corresponded to the official methods or methods available in the scientific literature for the analysis of crude and refined oils that were set up or adapted to AO and FAD analysis. Briefly, PV (expressed in milliequivalents of active oxygen per kg of fat) was determined by a volumetric titration in which the sample is dissolved in chloroform–acetic acid and treated with a solution of potassium iodide [40]. The *p*-AnV is a spectrophotometric method that measures a chromophore formed after the reaction in an iso-octane/acetic acid solution of the aldehydic compounds (mainly 2-alkenals and 2,4-alcadienals) present in the sample and the *p*-Anisidine [40]. POL (%) was determined by size molecular exclusion chromatography [40]. It was quantified by internal peak area normalization (%) considering the other lipid classes (triacylglycerols, diacylgycerols, monoacylglycerols, and FFA [40]). The evaluation of the oxidative stability by Rancimat (Metrohm, Herisau, Switzerland) at 120 °C is a measurement of its resistance to oxidation when it is subjected to air flow at a constant temperature. It consists of the measurement of the time elapsed until a rapid oxidation of the sample occurs (which agrees with a sudden jump in the conductivity in the vessel where the highly volatile oxidation products are collected), which is named as induction time (IT) [40]. The FA composition and tocol contents (α-, β-, γ-, and δ-T; and α-, β-, γ-, and δ-T3) were determined by GC-FID and by HPLC-FLD, respectively, as described in Varona et al. [40]: FA were quantified by internal peak area normalization (%), and tocols were quantified through calibration curves built with α-, β-, γ-, and δ-T, also applying the curves for each corresponding α-, β-, γ-, and δ-T3.

### 2.3. Statistics

As data did not follow a normal distribution (Shapiro–Wilk test, *p* ≤ 0.05), nonparametric tests were used for inferential analysis, and the mean, standard deviation, median, minimum, and maximum values were considered as descriptive statistical parameters, and boxplots were used to compare the distribution of the results between the groups. Mann–Whitney U-test was used to compare the PV, *p*-AnV, POL, IT, FA, T, T3, and T + T3 between AO and FAD sample groups (*n* = 92 for all variables, except for IT, for which 82 were included, as the measures of 10 AO samples did not provide a clear IT).

The distribution of each parameter for AO and for FAD samples from a similar botanical origin was described by using boxplot graphs. Kruskal–Wallis test was applied to AO samples and to FAD samples separately to determine if the variables were different between sample groups from different botanical origins, and the differences between groups were assessed by the stepwise multiple comparisons procedure of SPSS. In all cases, *p* ≤ 0.05 was considered significant. All univariate data analysis was performed with IBM SPSS Statistics (v 23, IBM, Armonk, NY, USA).

Principal component analysis (PCA) was conducted on AO and FAD as a multivariate data analysis, to explore the natural distribution and grouping of samples, to detect outlying samples, and to investigate correlations among variables. The data matrix used in this study consisted of 92 rows (samples) × 25 columns corresponding to the 25 variables: PV, *p*-AnV, POL, T, T3, C6:0, C8:0, C10:0, C12:0, C14:0, C16:0, C16:1 *n*-9, C16:1 *n*-7, C17:0, C18:0, *trans*-C18:1, C18:1 *n*-9, C18:1 *n*-7, C18:2 *n*-6, C20:0, C18:3 *n*-3, C20:1 *n*-9, C22:0, C23:0, and C24:0 (all variables were mean-centered and scaled to unit variance). The software used for PCA calculation was SIMCA v13.0 (Umetrics AB, Umea, Sweden). As IT could not be measured for all samples, it was not included in the PCA. Thus, its relationship with the rest of the variables was assessed through the Spearman’s correlation coefficients, calculated for AO (*n* = 69) and for FAD (*n* = 13) separately, considering *p* ≤ 0.05 as significant (IBM SPSS Statistics, v23, IBM, Armonk, NY). Significant correlation coefficients were graded as negligible (0.00–0.09), weak (0.10–0.39), moderate (0.40–0.69), strong (0.70–0.89), or very strong (0.90–1.00) according to the stratifications outlined by Schober et al. [41].

## 3. Results

### 3.1. Variability of the Oxidation Parameters and Differences between AO and FAD

All parameters significantly differed between AO and FAD, except for PV, *cis*-MUFA, and T3. Moreover, some of them showed a high variability in AO and FAD (Table 2).

FA composition and T contents were very variable, even within AO or FAD groups. Comparing the medians of both groups, SFA (C12:0, C14:0, and C16:0) were higher in FAD, while PUFA (C18:2 *n*-6 and C18:3 n-3) and T were higher in AO, resulting in a higher UFA/SFA in AO than in FAD (Table 2).

Primary oxidation measured by PV was low, both in the AO and FAD groups, with median values around 1 meq O_2_/kg of fat, even including samples with null values in both groups (Table 2). Secondary oxidation was assessed by measuring two parameters: *p*-AnV and POL. The amount of secondary oxidation aldehydes (measured through *p*-AnV, a parameter that principally determines 2-alkenals and 2,4-dienals) had significantly higher median values in the FAD group, while POL was higher in the AO group (Table 2). Indeed, POL were not detected in FAD. Regarding the oxidative stability measured by Rancimat at 120 °C (IT), globally, it was significantly higher in AO, reaching the highest maximum and median value compared to FAD, but its values varied in wider ranges than those of FAD samples (Table 2). Moreover, for some of the AO (10 samples), the IT could not be determined because the conductivity curve did not show a clear and sudden jump, even when the conductivity was measured for a long time, up to 200 h. Apart from this, it is remarkable the high variability observed for *p*-AnV and IT, both for AO and for FAD samples. In both groups, the IT was positively correlated with SFA and negatively correlated with UFA/SFA, both correlations being weak in AO and strong in FAD [41] (Table 3). Moreover, IT for AO was negatively correlated with POL and *trans*-C18:1 (weak correlation), while, for FAD, a significant negative correlation was observed with *p*-AnV (moderate correlation), *cis*-MUFA, and PUFA (both strong correlations) (Table 3).

### 3.2. Sample Clustering According to Botanical Origin

**Figure 1:**
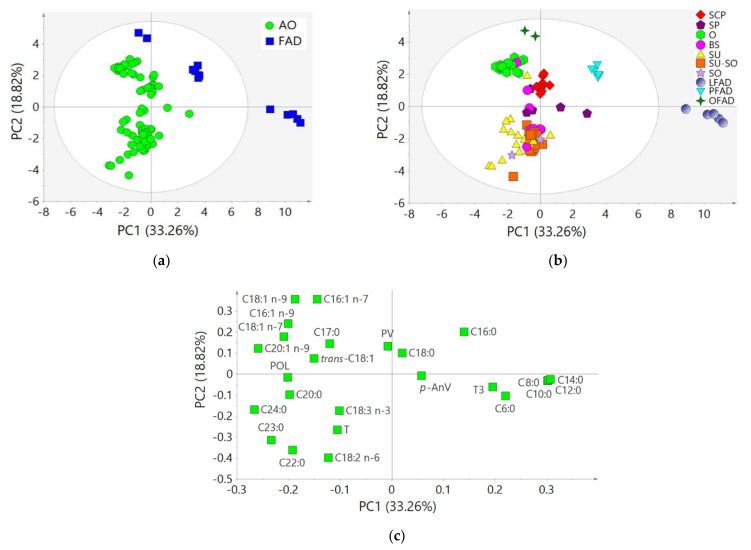
Principal component analysis (PCA) on the compositional and oxidation parameters (25 variables, mean-centered and scaled to unit variance) of acid oils from chemical refining (AO, *n* = 79) and fatty acid distillates from physical refining (FAD, *n* = 13). (**a**) Score plot colored according to the refining process, (**b**) score plot colored according to the botanical origin, and (**c**) loading plot. See Table 1 for abbreviations.

A PCA was conducted for AO (*n* = 79) and FAD (*n* = 13) samples to study natural clustering of samples, detecting possible outliers, and to reveal relationships between variables (Figure 1). The cumulative variance explained by the two first components was 52.1%.

The PCA score plot revealed a tendency of AO and FAD samples to cluster separately, and they were quite scattered (Figure 1a,b). By observing the loading plot (Figure 1c), it was evidenced that, in general, the separation of AO samples agreed with a high contribution of POL, T, some *cis*-MUFA, such as C16:1 n-7, C18:1 n-9, and C20:1 n-9, some *cis*-PUFA, such as C18:2 n-6 and C18:3 n-3, and long-chain SFA, such as C24:0 and C22:0. On the other hand, FAD were more related to *p*-AnV, some short- and medium-chain SFA (such as C6:0, C8:0, C10:0, C12:0, C14:0, and C16:0), and T3.

Furthermore, three different FAD clusters (corresponding to LFAD, PFAD, and OFAD group samples) and some AO clusters (corresponding to SCP and O group samples) were clearly distinguishable in the score plot (Figure 1). Short- and medium-chain FA and T3 contributed to the clustering of LFAD and C16:0 to that of PFAD. The two FAD samples plotted within the AO group corresponded to FAD from the refining of olive and olive pomace oils (OFAD). Indeed, the two OFAD samples laid close to the O cluster, and they all agreed with a high contribution of C18:1 n-9 and C16:1 n-7, while POL seemed to contribute more to O than to OFAD clustering (Figure 1). Other AO groups, such as SU or BS, were more scattered due to a high variability in their compositional parameters, especially due to the highly variable T (Figure 1).

Regarding the oxidation parameters, PV loadings were low, and basically no contribution to the explained variance was observed, especially for PC1. On the contrary, the *p*-AnV loadings were also relatively low but positive for PC1, contributing to the separation of the FAD group, while POL showed a high negative loading in PC1, contributing to the separation of some AO, especially of O and some SU (Figure 1).

### 3.3. Differences between Botanical Groups within AO and FAD

As the PCA revealed some clustering according to the botanical origin of the corresponding crude oils, we studied the differences in the compositional and oxidation parameters between the botanical groups within AO and FAD by using boxplot graphics.

#### 3.3.1. Oxidation and Oxidative Stability Parameters

**Figure 2:**
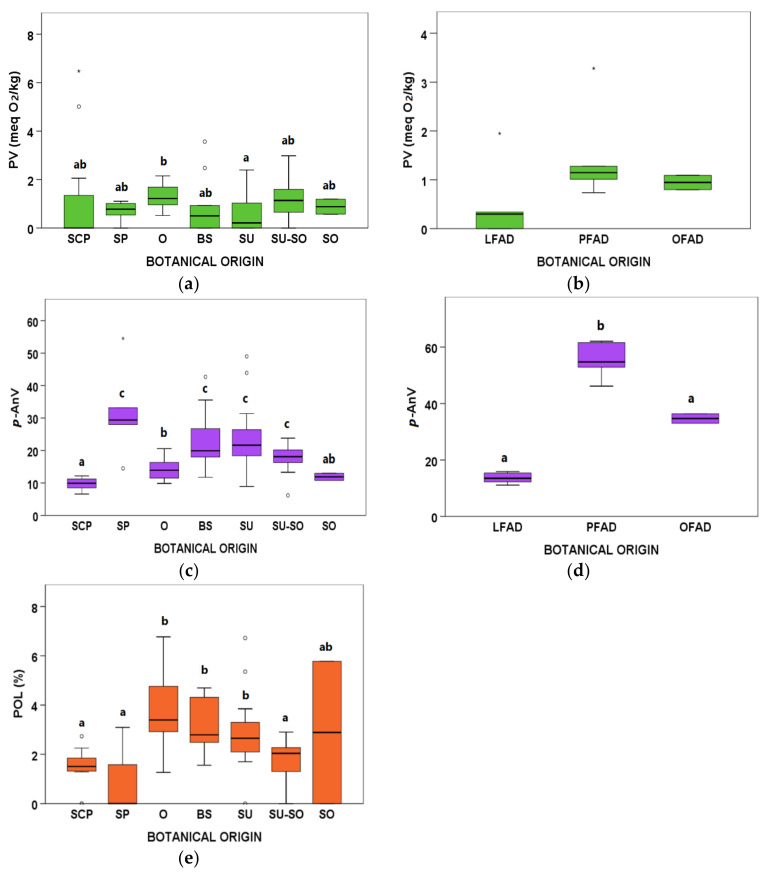
Peroxide value (PV), *p*-Anisidine value (*p*-AnV), and polymeric compounds (POL) boxplots according to botanical groups for (**a**,**c**,**e**) acid oils (AO) from chemical refining (*n* = 79), and (**b**,**d**) fatty acid distillates (FAD) from physical refining (*n* = 13) (see Table 1 for abbreviations of botanical groups). Within each type of byproduct (AO or FAD) and variable, botanical groups bearing different letters (a–c) show significantly different medians (*p* ≤ 0.05) according to Kruskal–Wallis test and stepwise multiple comparisons for independent samples. ° Outliers: samples with values between (Q3 + 1.5 × IQR) and (Q3 + 3 × IQR) or between (Q1 − 1.5 × IQR) and (Q1 − 3 × IQR). * Extreme outliers: samples with values above (Q3 + 3 × IQR) or below (Q1 − 3 × IQR). No POL were detected in FAD.

Regarding primary oxidation that was assessed by means of the PV, O presented the highest median value, while SU showed the lowest median (Figure 2a). Nevertheless, certain variability was observed in various groups, such as SCP, BS, SU, and SU-SO, where PV ranged from null to outlier or extreme values in some cases. No significant differences were observed for PV among FAD botanical groups, although LFAD tended to show lower values (Figure 2b).

Differences in *p*-AnV between AO botanical groups were also encountered, SP, BS, SU, and SU-SO being the groups with the highest median values, as well as the highest variability, while SCP showed the lowest median (Figure 2c). Outliers for *p*-AnV were found in the SP, BS, SU, and SU-SO groups. Regarding FAD, PFAD presented the highest and the most variable *p*-AnV values (Figure 2d).

With respect to POL, a high variability was observed within all the AO groups (Figure 2e). Moreover, SCP, SP, and SU-SO groups presented POL values significantly lower than O, BS, and SU groups. As commented above, no POL were detected in FAD samples.

**Figure 3:**
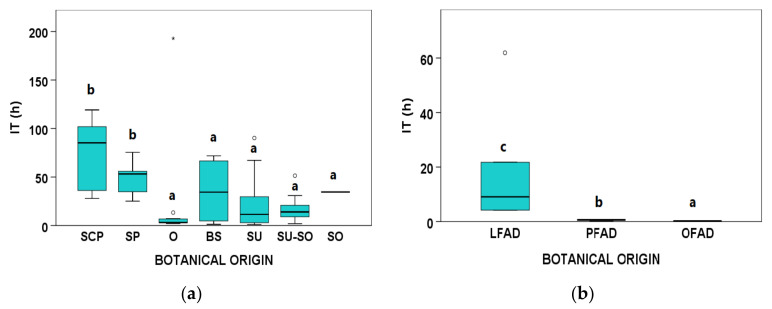
Boxplots for induction time (IT) measured by Rancimat at 120 °C according to botanical groups for (**a**) acid oils (AO) from chemical refining (*n* = 69, because in 10 AO samples the IT could not be determined) and (**b**) fatty acid distillates (FAD) from physical refining (*n* = 13) (see Table 1 for abbreviations of botanical groups). Within each type of byproduct (AO or FAD), botanical groups bearing different letters (a–c) show significantly different medians (*p* ≤ 0.05) according to Kruskal–Wallis test and stepwise multiple comparisons for independent samples. ° Outliers: samples with values between (Q3 + 1.5 × IQR) and (Q3 + 3 × IQR) or between (Q1 − 1.5 × IQR) and (Q1 − 3 × IQR). * Extreme outliers: samples with values above (Q3 + 3 × IQR) or below (Q1 − 3 × IQR).

Regarding the oxidative stability (IT) measured by Rancimat at 120 °C, significant differences were observed between botanical origin groups from both AO and FAD (Figure 3). In AO, the most stable samples were SCP and SP, but, in general, a high variability was observed, including some outliers in O, SU, and SU-SO (Figure 3a). Regarding FAD, the IT values observed for LFAD were higher and more variable than those of PFAD and OFAD (Figure 3b).

#### 3.3.2. Fatty Acid (FA) Composition

**Figure 4:**
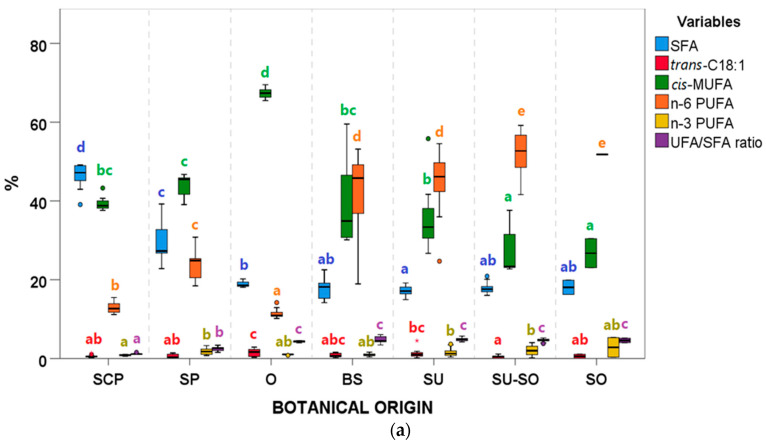

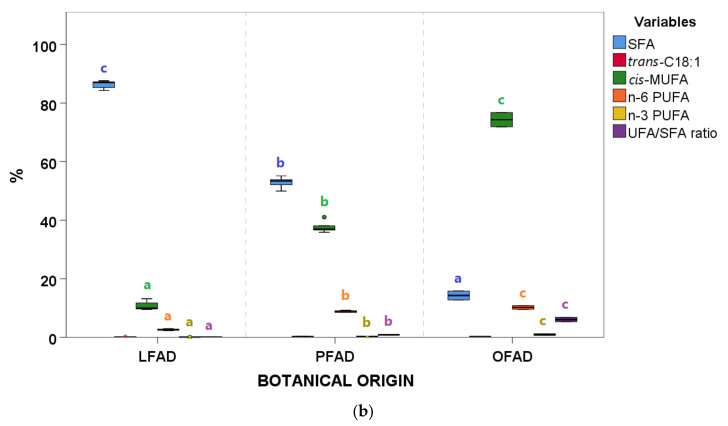
Saturated fatty acids (SFA), trans-C18:1, cis-monounsaturated fatty acids (cis-MUFA), polyunsaturated fatty acids (n-6 and n-3 PUFA), and boxplots according to botanical groups for (**a**) acid oils (AO) from chemical refining (*n* = 79) and (**b**) fatty acid distillates (FAD) from physical refining (*n* = 13) (see Table 1 for abbreviations of botanical groups). Within each type of byproduct (AO or FAD) and variable, botanical groups bearing different letters (a–e) show significantly different medians (*p* ≤ 0.05) according to Kruskal–Wallis test and stepwise multiple comparisons for independent samples. Tables S2 and S3 in the Supplementary Materials report the numeric results of the FA composition. ° Outliers: samples with values between (Q3 + 1.5 × IQR) and (Q3 + 3 × IQR) or between (Q1 − 1.5 × IQR) and (Q1 − 3 × IQR).

The variations in SFA, MUFA, and PUFA between botanical groups were reported in Varona et al. [1]. Here, the detailed FA composition is reported, including n-6 and n-3 PUFA, as they are substrates of lipid oxidation (Figure 4 and Tables S1–S3 in Supplementary Materials).

Briefly, regarding FA composition within AO, the SCP group was the richest in SFA, followed by SP (which, besides, showed the highest variability), while SU showed the lowest SFA median value (Figure 4a). Considering *trans*-C18:1 % in AO, the O group showed the highest values. The highest MUFA % was observed in O samples, followed by SP, SCP, and BS, which contained palm oil in their blends. n-6 PUFA medians in SU-SO and SO groups were the highest, followed by SU and BS, with all these groups (except for SO) presenting a high variability. Considering n-3 PUFA %, the highest values were found in SU-SO, SU, and SP groups. Overall, the UFA/SFA ratio was only significantly different for SCP and SP groups, which showed the lowest ratios.

Within FAD, differences between FA composition were also observed but, in this case, the intragroup variabilities were lower. The lowest SFA median was found in OFAD group (Figure 4b). No differences were observed for *trans*-C18:1 medians (0.15% for LFAD, 0.21% for PFAD, and 0.22% for OFAD), while OFAD group presented the highest % of *cis*-MUFA, n-6 PUFA, and n-3 PUFA, followed by PFAD. Thus, the UFA/SFA ratio also showed the highest values in OFAD samples, followed by PFAD, and then by LFAD.

#### 3.3.3. Tocopherol (T) and Tocotrienol (T3) Content

**Figure 5:**
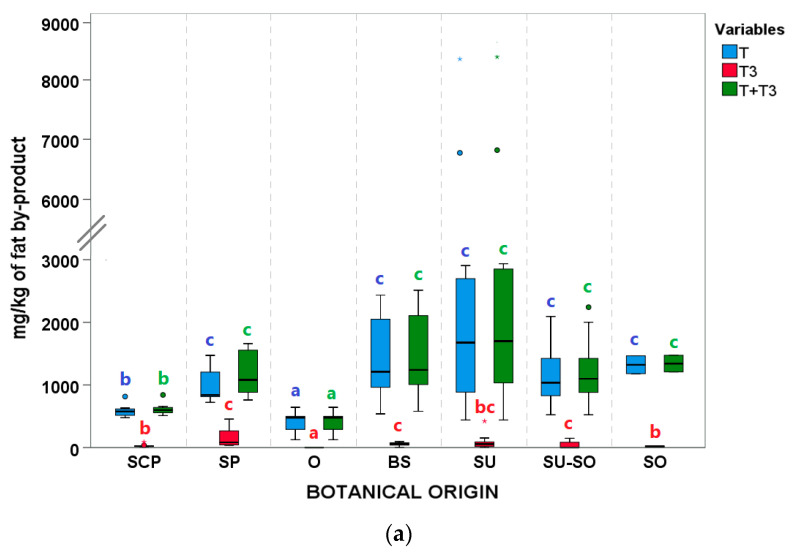

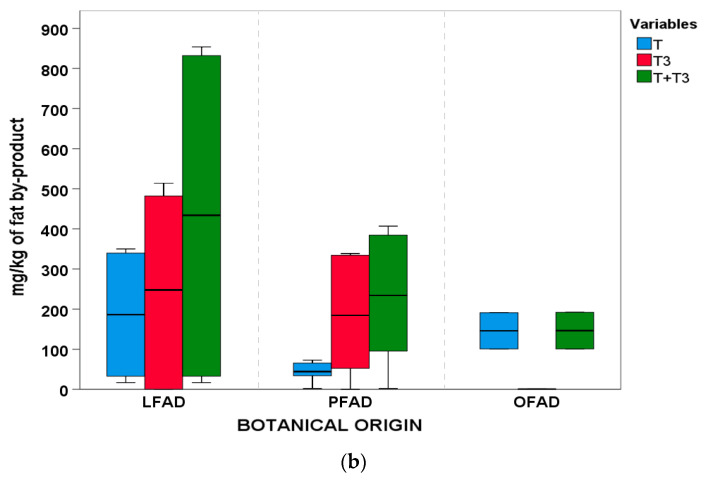
Tocopherols (T), tocotrienols (T3), and T + T3 contents according to botanical groups for (**a**) acid oils (AO) from chemical refining (*n* = 79) and (**b**) fatty acid distillates (FAD) from physical refining (*n* = 13) (see Table 1 for abbreviations of botanical groups). Within each type of byproduct (AO or FAD) and variable, botanical groups bearing different letters (a–c) show significantly different medians (*p* ≤ 0.05) according to Kruskal–Wallis test and stepwise multiple comparisons for independent samples. ° Outliers: samples with values between (Q3 + 1.5 × IQR) and (Q3 + 3 × IQR) or between (Q1 − 1.5 × IQR) and (Q1 − 3 × IQR). * Extreme outliers: samples with values above (Q3 + 3 × IQR) or below (Q1 − 3 × IQR).

Considering AO, differences were observed for the content of tocols depending on the botanical origin (Figure 5a). The lowest T content was found for samples from the refining of olive and olive pomace oils, closely followed by SCP blends. They both also presented T values that varied within narrow ranges. The rest of botanical groups presented higher T values. Interestingly, two SU samples presented extreme T values (6694.9 and 8464.4 mg/kg).

About the global sum of T + T3 in AO, it showed a similar profile to T, as the sum of tocopherols (T) was the main contributor to the sum of T + T3 (Figure 5a). Regarding FAD, no significant differences between medians were observed between botanical origins for T, nor for T3 (Figure 5b).

## 4. Discussion

The results of this study revealed a high variability in the content of oxidation compounds and in the oxidative stability of AO and FAD, even within samples from similar origins. This fact is in addition to the high variability also encountered for their energy value [1], reflecting a lack of standardization of these feed fat ingredients.

Overall, primary oxidation (assessed by means of the PV) was low in all samples, and no relationship with the refining process or botanical origin could be clearly defined. Regarding secondary oxidation, *p*-AnV and POL were clearly influenced by the type of refining, rather than by the botanical origin of the crude oil, the aldehydes content being higher in FAD and the POL content higher in AO. On the contrary, T and T3 contents and FA composition were more related to the botanical origin of the crude oils.

### 4.1. Influence of the Refining Process (AO vs. FAD)

First, regarding primary oxidation, no differences were encountered between AO and FAD groups, and neither of them presented samples with high PV, which agreed with those results previously found by Nuchi et al. [19] for similar byproducts. Moreover, Kuntom et al. [42] found PV values in PFAD below 5 meq O_2_/kg in most samples, except for two outliers with PV above 10 meq O_2_/kg. As a primary oxidation parameter, PV measures lipid hydroperoxides, which are unstable at high temperatures. In the case of chemical refining, temperatures reached during the neutralization step, centrifugation, and acidification to obtain AO usually rise to 80–90 °C, while, during the deodorization step in physical refining, higher temperatures (180–270 °C) and low pressure (0.05–0.8 kPa) are applied to remove FFA by distillation [21,43]. Therefore, it can be expected that AO and FAD samples that are recently produced might show PV close to 0 due to peroxide decomposition at the temperatures reached during their obtention. Once AO and FAD are obtained, PV will start to increase during storage, the rate and magnitude of the increase being dependent on the oxidizability of the sample and storage conditions.

However, even if, in these AO and FAD, the PV values were low, the oxidation degree was high in some of them due to the presence of secondary oxidation compounds assessed by both *p*-AnV and POL. Secondary oxidation compounds in these byproducts might originate by peroxide decomposition during sample storage, but they can also come from the initial crude oils (to a greater or lesser extent, depending on their degree of oxidation) or be formed during the heat treatments applied during the refining of the crude oils and the obtaining of the AO and FAD. Regarding *p*-AnV, wide ranges were found in both AO and FAD, as also reported by Nuchi et al. [19]. The *p*-AnV is a parameter that is particularly sensible to 2-alkenals and 2,4-alcadienals, which are easily formed from the breakdown of peroxides from PUFA, such as linolenic and linoleic acids. Although these PUFA were mainly present in AO, the *p*-AnV values in FAD samples were surprisingly higher. This was found also in OFAD compared to O, even though both were obtained from olive oils, but, as only two OFAD samples were included, this comparison is somehow tentative. The highest *p*-AnV found in FAD agreed with the higher temperatures usually applied in the physical refining process [27,43]. Moreover, since FAD are obtained by distillation, during this process, the secondary oxidation aldehydes will also be distilled and accumulated in FAD together with other distillable compounds, such as FFA and T [44,45,46]. On the contrary, high molecular weight compounds, such as POL, might not be distilled at all, or at very low amounts, explaining the null POL contents found in the FAD samples. However, in AO, POL might be washed out from the oil and accumulated in AO after the separation of the soap stocks in the neutralization step. The higher POL content in AO compared to FAD was also reflected when the two OFAD samples were compared with the O samples. POL mainly consist of triacylglycerol polymers created by the reaction of triacylglycerol radicals and accumulated in the advanced stages of oxidation [2]. In general, the formation of POL is higher in oils with higher PUFA contents, because they are more prone to form radicals [47]. In fact, SCP and SP, as the more saturated groups in AO, presented POL values that were significantly lower than the rest, whereas, remarkedly, SU-SO also showed significant lower values, despite this group being more unsaturated.

### 4.2. Influence of the Botanical Origin of the Crude Oil

The FA and tocol composition of crude oils influence the FA composition and T + T3 contents of these refining byproducts. According to Varona et al. [1], the significant differences found for FA and tocols when AO and FAD were compared could be explained by the fact that the type of refining (chemical or physical) is usually selected according to the composition of the crude oil, which depends on its botanical origin. As more drastic conditions are usually applied in physical refining, it is preferred for more saturated crude oils, such as coconut, palm kernel, and palm oils, obtaining FAD with FA and tocol profiles typical of these fats. On the contrary, chemical refining is usually preferred for crude oils rich in unsaturated FA, which are more prone to oxidation [44,45,46]. Therefore, the FA composition of AO is usually richer in PUFA, such as C18:2 n-6 and C18:3 n-3, and in T, reflecting the FA and T profile of the unsaturated crude oils (soybean, sunflower, grapeseed, corn oils, etc.) [48]. This influence of the botanical origin of the crude oil was also evident even within different types of AO and FAD, as reported in Varona et al. [1]: the SU-SO and SO groups, followed by SU and BS, were the richest in n-6 PUFA (mainly represented by C18:2 n-6) and in T, as they come from seed oils; the O and OFAD groups, followed by the groups containing palm oil byproducts (SCP, SP, PFAD), were the richest in C18:1 n-9, as they come from olive or palm oils; PFAD were the richest FAD in palmitic acid and in T3, as they are byproducts from palm oil refining; SCP blends were the richest AO in palmitic and stearic acids, as SCP included AO from the refining of cocoa butter (which is rich in C16:0 and C18:0); and LFAD showed a high content of medium-chain SFA, which are typical FA in coconut and palm kernel oils [23,48] (see also Table S1, Supplementary Material). The presence of corn and palm AO in SP and SCP blends was also reflected in their γ-T3 content. In addition, as discussed in Varona et al. [1], the two samples in the SU group with extremely high T contents could agree with the addition of tocol-rich products, such as deodistillates obtained from the deodorization step in chemical refining.

Thus, the botanical origin determines the final FA and T composition of AO and FAD, which could have a role in the oxidative stability (IT) of these byproducts. However, even though, globally, AO presented higher IT medians than FAD, this depended on the botanical group, as different IT medians were observed between various botanical groups. Knowing that FA oxidizability mainly depends on their degree of unsaturation, in order to relate the IT with the FA composition, the UFA/SFA ratio was calculated, including all unsaturated FA in UFA and all saturated FA in SFA (regardless of the configuration of the double bond). Thus, this calculation is slightly different from the U/S ratio usually used to predict the energy value of fats and that was reported in Varona et al. [1], in which SFA with 12 carbons or below were considered as unsaturated fatty acids (thus, in the numerator –U- of that ratio), and *trans*-FA were considered as SFA (thus, in the denominator –S- of the ratio). The botanical group with the lowest UFA/SFA median was SCP, which also led to the highest IT median, even if it was the group with the lower T content. Then, it was followed by SP group and by AO groups, such as SU and SU-SO, that presented higher UFA/SFA. This resulted in a negative correlation between IT and UFA/SFA for AO. The FA composition influence could also explain the negative correlation between IT and POL in AO, as it agreed with a concomitant negative correlation between POL and SFA; since SFA are less prone to oxidation, they might have not only led to higher IT, but also to lower POL formation.

Nevertheless, some of the AO groups presented longer IT than the FAD groups, even if AO were richer in n-6 and n-3 PUFA that are more prone to lipid oxidation reactions. This could be explained by the higher T content in AO that would be acting as an antioxidant, delaying the onset of oxidation of these AO. Moreover, the addition of synthetic antioxidants to some AO could not be excluded and would explain some of the extremely high IT values found for certain AO. In fact, the sample suppliers reported the addition of synthetic antioxidants in 16 AO samples, while they did not do so in any of the FAD samples.

The T and T3 content of these byproducts was highly variable, even within botanical groups [1]. Interestingly, two SU samples deserved special attention as they were revealed as outliers in the boxplot. Apart from the very high T amounts (8464.39 and 6694.94 mg/kg) that they presented, they also had very high *p*-AnV (49.0 and 43.9, respectively). Actually, these *p*-AnV were more in the range of *p*-AnV found for FAD samples than those found for AO, which supports the hypothesis of the possible addition of deodistillates from the deodorization step to these AO [1]. In AO samples, no significant correlation (*p* = 0.326) between T content (major tocols) and IT was observed, which could be related to the addition of synthetic antioxidants in some samples. In contrast, in FAD samples, where the suppliers did not report antioxidant addition, an almost significant correlation (*p* = 0.071) was observed between T3 content (major tocols) and IT.

### 4.3. Comparison with Fat Quality Thresholds in EU Regulations and Guidelines

EU regulations regarding feed ingredients do not include any parameter to evaluate the oxidation status of feed fats, nor to assess primary or secondary oxidation [20]. Some animal nutrition associations include PV assessment in their guidelines for fat sources used in feeds, setting its threshold at 10 meq O_2_/kg for various AO and FAD [36]. In the present study, no AO or FAD samples were above this limit. However, since peroxides can decompose into secondary oxidation compounds or radicals can form POL [2,49], the measurement of PV is totally insufficient as a global evaluation of fat oxidation and should be evaluated together with a secondary oxidation parameter [2,19,38]. However, the evaluation of secondary oxidation is not usually included in the quality guidelines [36], nor routinely performed by the producers.

The type of secondary oxidation products formed depends on the FA composition of the fat, as well as on the oxidation conditions (O_2_ availability, temperature, light exposure). Thus, the parameter to evaluate the secondary oxidation in a feed fat should be accordingly selected to obtain a realistic evaluation of the oxidation status. In this study, we have chosen the *p*-AnV, as it is especially adequate to evaluate 2-alkenals and 2,4-alcadienals that are characteristic breakdown products of lipid hydroperoxides formed from C18:2 n-6, C18:3 n-3, and more unsaturated FA, and at a lesser extent, from C18:1 n-9 [49,50]. Therefore, it is a parameter suitable for most vegetable oils and for some animal fats, and has been successfully applied to feed fat ingredients [19,38]. In other studies, TBA value has been selected instead of *p*-AnV [51]. However, TBA value mainly reacts with malondialdehyde, which is especially formed from PUFA with more than two double bonds, and not from MUFA [4,38]. According to this, Nuchi et al. [19] reported a good application of TBA value for fish oils, with it not being sensitive enough to evaluate secondary oxidation of the rest of feed fat byproducts, for which *p*-AnV was preferred. Moreover, considering that *p*-AnV is a simple and rapid analytical determination, it could be a suitable parameter to be included in feed quality control guidelines for vegetable fats and oils. The values obtained in this study were very similar to those reported by Nuchi et al. [19] for AO and FAD. However, unfortunately, since it is not currently included in the guidelines, the results of this study cannot be compared with an established threshold. In fact, comparing the absolute values of this index to draw conclusions about the degree of oxidation of different fats would only make sense when fats have a similar FA composition, since the formation of aldehydes depends on that.

Regarding POL, it is neither included in feed fat regulations or guidelines. Even its determination is not as simple and economic as that of *p*-AnV; it has been suggested as a complementary control parameter for feed fats [19]. It would provide additional information to that of *p*-AnV or TBA, and, moreover, as it has been commented above, POL have been related with digestibility effects. However, as FAD have shown not to accumulate POL, its determination would not be a priority for this type of fat byproduct. However, POL content has been suggested to control the feed fats submitted for long heating periods to high temperatures [19,38], such as recycled cooking oils (if permitted in feeds). In AO, the POL content correlated positively with the UFA/SFA ratio (*p* = 0.016) and negatively with the IT (*p* = 0.002). Thus, this parameter could also be interesting for the quality control of AO.

Overall, considering the repercussions described above for the occurrence of oxidation in feed fats, the evaluation of fat oxidation should be included in a global evaluation of feed fat quality, including proper primary and secondary parameters. Unfortunately, currently this is not the case in regulations, feed fat quality guidelines, or tables on the chemical composition and nutritional value of feedstuff [35,36]. As a consequence, some producers usually do not report these values in the products’ technical sheets, or they only report PV, lacking a complete information of the oxidative status of the fat. The high variability in the oxidation status observed in this study for AO and FAD, sometimes even within batches from the same producer, supports the inclusion of these parameters in the routine characterization of the products. Their values might depend on the composition of the crude oil, the refining and byproduct obtention processes, and storage time and conditions. Any efforts towards the reduction in the global oxidation, or at least towards its standardization, would contribute to increasing the farmers’ confidence in them, as was already recommended for the parameters determining the energy value of the fats [1]. In this respect, various actions could be considered, ranging from the standardization of the byproduct collection in the waste tank by avoiding mixing AO or FAD from various sources to the optimization of the distillation step in physical refining by adjusting the time, temperature, and stripping agent, or the post-treatment of AO and FAD to standardize their composition or reduce their oxidation compounds.

## 5. Conclusions

AO and FAD from the refining of edible oils and fats provide energy, liposoluble vitamins, and essential nutrients to an animal diet. Results from this study have shown that the oxidative status and oxidative stability of these byproducts can be highly variable, even between samples from similar botanical origins. Since oxidation aldehydes are easily distillable in the deodorization step, FAD presented higher *p*-AnV, while AO presented higher POL. Primary oxidation was low, both in AO and FAD, due to the instability of peroxides at the high temperatures usually reached during the obtention of these byproducts. Increases in PV would be the result of the storage conditions over time. Moreover, wide ranges for some of the studied oxidation parameters were observed even when the AO or the FAD had been obtained from the same type of crude oils. The botanical origin of the crude oil also affected the FA and the tocol contents. All of these variations led to highly variable oxidative stability for all types of samples. Therefore, the inclusion of adequate primary and secondary oxidation parameters in the quality control of these byproducts is necessary to guarantee a constant composition and stability and, thus, proper quality. This could contribute to farmers obtaining more consistent production results when animals are fed these fats, which is essential to upcycle and to valorize these byproducts.

## Figures and Tables

**Table 1:** Samples’ classification according to the refining process and botanical origin.

Refining Process	Group ^1^	Botanical Origin	Subgroup: Different Mixtures	*N*	Total
Chemical refining (Acid oils, AO)	SCP	Blends of AO from seed oils, cocoa Butter, and palm oil	Cocoa butter, rapeseed, soybean, and palm oils (40/30/20/10)	2	12
			Cocoa butter, palm, and seed oils ^2^	10	
	SP	Blends of AO from seed and palm oils	Soybean, rapeseed, and palm oils (40/40/20)	2	5
			Sunflower, soybean, palm, corn, and rapeseed oils ^2^	3	
	O	AO from olive pomace oil and blends of AO from olive pomace and olive oils	Olive pomace oil	13	18
			Olive pomace and olive oils (90/10)	5	
	BS	Blends of AO from seed oils	Sunflower (80–90), rapeseed (20–10), and traces of palm and palm kernel oils and palm stearin ^3^	1	9
			Sunflower, corn, and grapeseed oils (40/30/30)	3	
			Sunflower, soybean, and corn oils ^2^	3	
			Sunflower, high oleic sunflower, soybean, corn, and olive pomace oils ^2^	2	
	SU	AO from sunflower oil	Sunflower oil	18	18
	SU-SO	Blends of AO from sunflower and soybean oils	Sunflower and soybean oils ^2^	4	15
			Sunflower and soybean oils (10/90)	7	
			Sunflower and soybean oils (80/20)	2	
			Sunflower and soybean oils (90/10)	2	
	SO	AO from soybean oil	Soybean oil	2	2
Physical refining (Fatty acid distillates, FAD)	LFAD	FAD from coconut oil and blends of FAD from coconut and palm kernel oils (lauric oils)	Coconut oil	2	5
			Coconut and palm kernel oils ^2^	3	
	PFAD	FAD from palm oil	Palm oil	6	6
	OFAD	FAD from olive pomace and olive oils	Olive pomace oil	1	2
			Olive oil	1	

^1^ See the “Group” and “Botanical origin” columns for the definition of abbreviations. ^2^ For some blends the proportions were unknown. ^3^ Some blends have traces of fruit oils.

**Table 2:** Mean, standard deviation, median, minimum, and maximum values obtained for acid oils (AO) from chemical refining and fatty acid distillates (FAD) from physical refining.

Parameter	Acid Oils (AO, *n* = 79)				Fatty Acid Distillates (FAD, *n* = 13)				*p* ^1^
	Mean ± SD	Median	Min	Max	Mean ± SD	Median	Min	Max	
C12:0 (%)	0.1 ± 0.91	0.0	0.0	8.1	13.1 ± 17.07	0.3	0.0	36.4	<0.001
C14:0 (%)	0.2 ± 0.40	0.1	0.1	3.6	8.0 ± 9.63	1.2	0.0	24.0	<0.001
C16:0 (%)	13.6 ± 4.14	13.0	8.1	25.2	29.1 ± 16.44	18.9	10.3	47.5	0.001
C18:0 (%)	6.9 ± 6.93	3.9	2.3	25.0	4.5 ± 1.74	4.5	1.8	8.2	0.594
C18:1 n-9 (%)	40.5 ± 14.74	36.6	21.1	67.1	31.7 ± 21.05	35.7	9.3	71.6	0.173
C18:2 n-6 (%)	32.1 ± 18.07	36.8	10.2	59.2	6.6 ± 3.37	8.5	2.3	10.8	<0.001
C18:3 n-3 (%)	1.4 ± 1.03	1.1	0.2	5.3	0.3 ± 0.32	0.3	0.0	1.2	<0.001
SFA (%) ^2^	23.0 ± 10.62	18.5	14.2	49.1	59.8 ± 25.74	53.8	12.8	87.6	<0.001
*cis*-MUFA (%) ^2^	42.5 ± 15.31	38.1	22.7	69.5	33.0 ± 22.43	36.7	9.6	76.8	0.121
*trans*-C18:1 (%) ^2^	0.9 ± 0.82	0.7	0.0	4.5	0.2 ± 0.10	0.2	0.1	0.5	<0.001
n-6 PUFA (%) ^2^	32.2 ± 18.05	36.8	10.2	59.2	6.7 ± 3.39	8.5	2.3	10.9	<0.001
n-3 PUFA (%) ^2^	1.4 ± 1.03	1.1	0.2	5.3	0.3 ± 0.32	0.3	0.0	1.2	<0.001
UFA/SFA ratio ^3^	4.0 ± 1.38	4.4	1.0	6.1	1.4 ± 2.13	0.9	0.1	6.8	<0.001
T (mg/kg)	1167.1 ± 1234.52	813.1	126.1	8464.4	113.8 ± 117.93	65.6	1.8	350.0	<0.001
T3 (mg/kg)	48.3 ± 80.21	21.8	0.0	454.7	179.9 ± 194.13	89.3	0.0	513.9	0.065
T + T3 (mg/kg)	1215.4 ± 1247.57	840.7	126.1	8514.0	293.7 ± 287.64	192.0	1.8	853.7	<0.001
PV (meq O_2_/kg)	1.0 ± 1.12	0.8	0.0	6.5	1.0 ± 0.88	1.0	0.0	3.3	0.791
*p*-AnV	18.4 ± 9.39	16.4	6.2	54.5	36.1 ± 20.31	36.4	11.1	62.1	0.006
POL (%) ^4^	2.6 ± 1.58	2.5	ND	6.8	ND	ND	ND	ND	<0.001
IT (h) ^5^	31.1 ± 36.83	20.5	1.0	192.9	8.1 ± 17.26	0.7	0.3	61.9	<0.001

Abbreviations: SFA, saturated fatty acids; *cis*-MUFA, monounsaturated fatty acids; PUFA, polyunsaturated fatty acids; UFA/SFA ratio, unsaturated/saturated ratio; T, sum of α-, β-, γ-, and δ-Tocopherol; T3, sum of α-, β-, γ-, and δ-Tocotrienol; T + T3, sum of tocopherols and tocotrienols; PV, peroxide value; *p*-AnV, *p*-Anisidine value; POL, polymeric compounds; IT, induction time; SD, standard deviation; ND, not detected. ^1^ *p* values were obtained from Mann–Whitney U-test for independent samples to compare medians between AO and FADs. *p* ≤ 0.05 was considered significant. ^2^ Table shows the sums of fatty acids, including all the identified and quantified FA, expressed as internal area normalization in %. SFA includes: C6:0, C8:0, C10:0, C11:0, C12:0, C13:0, C14:0, C15:0, C16:0, C17:0, C18:0, C20:0, C21:0, C22:0, C23:0, and C24:0; cis-MUFA includes C16:1 n-9, C16:1 n-7, C18:1 n-9, C18:1 n-7, and C20:1 n-9; n-6 PUFA includes C18:2 n-6 and C20:2 n-6; n-3 PUFA includes C18:3 n-3; *trans*-C18:1 includes a sum of positional isomers. ^3^ To calculate the UFA/SFA ratio, the following relationship was considered: the sum of *cis*-MUFA, *cis*-PUFA, and *trans*-C18:1/ SFA. ^4^ POL was quantified by internal peak area normalization in % (in relation to the sum of the peak areas of all lipid classes POL, triacylglycerols, diacylglycerols, monoacylglycerols, and free fatty acids). In FAD, *n* = 8 for POL, as lipid classes could not be determined in the lauric FAD samples. ^5^ In AO, *n* = 69 for IT, as IT value could not be determined for 10 AO samples.

**Table 3:** Spearman’s correlation coefficients (Spearman’s Rho) between the induction time (IT) and the main variables determined in acid oils (AO) from chemical refining and in fatty acid distillates (FAD) from physical refining.

Parameter	AO (*n* = 69) ^1^	FAD (*n* = 13)
PV	NS	NS
*p*-AnV	NS	–0.577 *
POL	–0.375 **	NA
SFA	0.507 **	0.819 **
*cis*-MUFA	NS	–0.819 **
*trans*-C18:1	–0.264 *	NS
n-6 PUFA	NS	–0.758 **
n-3 PUFA	NS	–0.830 **
UFA/SFA ratio	–0.507 **	–0.819 **
T	NS	NS
T3	NS	NS
T + T3	NS	NS

See Table 2 for abbreviations; NA, not available, because the content of POL was null in FAD. ^1^ For 10 AO samples, no IT could be determined. NS, not significant coefficient (*p* > 0.05). * Statistically significant coefficient (*p* ≤ 0.05). ** Statistically significant coefficient (*p* ≤ 0.01).

## Data Availability

Not applicable.

## Supplementary Materials

The following are available online https://www.mdpi.com/article/10.3390/ani11092559/s1, Table S1: Median values for the individual fatty acids according to the type of by-product and the botanical origin; Table S2: Mean, standard deviation, median, minimum and maximum values for the different sums of fatty acids identified and quantified by peak area normalization (%) according to the botanical origin of the acid oils (AO); Table S3: Mean, standard deviation, median, minimum and maximum values for the different sums of fatty acids identified and quantified by peak area normalization (%) according to the botanical origin of the fatty acid distillates (FAD).
