# Supplementary material for: Oxidative Quality of Acid Oils and Fatty Acid Distillates Used in Animal Feeding
Source: Animals (Basel). 2021 Aug 31;11(9):2559. doi: 10.3390/ani11092559 (PMC8467598; doi:10.3390/ani11092559)
Supplement: Supplementary file 1 [file animals-11-02559-s001.zip › animals-1326190-supplementary.pdf]

SUPPLEMENTARY MATERIAL

Table S1. Median values for the individual fatty acids identified and quantified by internal normalization (peak area percentage) according to the type of by-product and the botanical origin.

By-product	Botanical origin	C6:0	C8:0	C10:0	C12:0	C14:0	C16:0	C16:1 n-9	C16:1 n-7	C17:0	C18:0	C18:1 *trans*	C18:1 n-9	C18:1 n-7	C18:2 n-6	C20:0	C18:3 n-3	C20:1 n-9	C20:2 n-6	C22:0	C22:2	C24:0
Acid oils (AO, *n* = 79)	SCP	0.00^{a}	0.01^{a}	0.01^{a}	0.02^{bc}	0.15^{c}	21.86^{d}	0.05^{b}	0.35^{b}	0.20^{c}	23.38^{c}	0.48^{ab}	37.26^{c}	0.96^{a}	12.67^{b}	0.98^{c}	0.90^{a}	0.18^{a}	0.00^{ab}	0.41^{a}	0.00	0.29^{a}
	SP	0.02^{b}	0.09^{c}	0.04^{c}	0.25^{d}	0.38^{c}	18.25^{c}	0.05^{ab}	0.17^{a}	0.10^{ab}	5.34^{ab}	0.31^{ab}	43.94^{c}	1.24^{b}	24.87^{c}	0.45^{a}	1.77^{b}	0.28^{b}	0.00^{a}	0.47^{b}	0.00	0.31^{ab}
	O	0.01^{b}	0.02^{bc}	0.01^{b}	0.01^{a}	0.06^{a}	13.20^{b}	0.13^{c}	0.75^{c}	0.11^{b}	3.63^{a}	1.66^{c}	64.14^{d}	1.95^{c}	10.83^{a}	0.56^{a}	1.05^{ab}	0.32^{c}	0.09^{b}	0.40^{a}	0.00	0.55^{c}
	BS	0.00^{ab}	0.03^{bc}	0.02^{bc}	0.02^{d}	0.10^{b}	13.06^{ab}	0.06^{b}	0.16^{a}	0.09^{a}	3.41^{ab}	0.66^{abc}	33.24^{bc}	1.04^{ab}	45.81^{d}	0.54^{a}	0.90^{ab}	0.24^{b}	0.00^{ab}	0.66^{bc}	0.00	0.42^{bc}
	SU	0.07^{b}	0.03^{c}	0.02^{c}	0.03^{cd}	0.12^{b}	9.81^{a}	0.07^{b}	0.19^{a}	0.09^{a}	3.87^{ab}	1.01^{bc}	31.66^{b}	1.20^{b}	46.14^{d}	0.79^{b}	1.26^{b}	0.25^{b}	0.00^{ab}	0.93^{d}	0.00	0.50^{c}
	SU-SO	0.00^{ab}	0.02^{bc}	0.01^{ab}	0.01^{ab}	0.11^{b}	11.44^{a}	0.03^{a}	0.12^{a}	0.10^{ab}	4.07^{b}	0.44^{a}	22.36^{a}	1.14^{b}	52.70^{e}	0.54^{a}	1.99^{b}	0.19^{a}	0.00^{a}	0.80^{cd}	0.00	0.44^{bc}
	SO	0.00^{ab}	0.01^{ab}	0.01^{ab}	0.01^{a}	0.10^{b}	11.51^{ab}	0.03^{a}	0.11^{a}	0.09^{ab}	4.09^{ab}	0.55^{ab}	25.17^{a}	1.24^{ab}	51.79^{e}	0.68^{ab}	2.83^{b}	0.19^{ab}	0.01^{ab}	0.93^{cd}	0.00	0.45^{bc}
p values [1]		0.010	0.000	0.000	0.000	0.000	0.000	0.000	0.000	0.000	0.000	0.001	0.000	0.000	0.000	0.000	0.021	0.000	0.006	0.000	0.108	0.000
Fatty acid distillates (FAD, *n* = 13)	LFAD	0.53^{b}	6.60^{b}	5.55^{c}	33.50^{c}	18.25^{c}	15.92^{a}	0.01^{a}	0.06^{a}	0.03^{a}	4.58	0.15	9.58^{a}	0.18^{a}	2.52^{a}	0.11^{a}	0.08^{a}	0.05^{a}	0.00	0.03^{a}	0.00	0.02^{a}
	PFAD	0.00^{a}	0.03^{a}	0.02^{b}	0.22^{b}	1.16^{b}	46.31^{b}	0.03^{b}	0.16^{b}	0.11^{b}	4.53	0.21	35.98^{b}	0.64^{b}	8.93^{b}	0.34^{b}	0.35^{b}	0.13^{b}	0.01	0.06^{a}	0.00	0.04^{ab}
	OFAD	0.00^{a}	0.01^{a}	0.00^{a}	0.01^{a}	0.05^{a}	11.78^{a}	0.14^{c}	1.31^{c}	0.07^{a}	1.86	0.22	69.75^{c}	2.83^{c}	10.13^{c}	0.29^{ab}	0.95^{c}	0.29^{c}	0.09	0.10^{b}	0.00	0.05^{b}
p values [2]		0.004	0.008	0.006	0.006	0.006	0.008	0.006	0.006	0.006	0.094	0.493	0.006	0.006	0.006	0.011	0.006	0.006	0.056	0.036	1.000	0.049
AO (*n* = 79)		0.00	0.02^{a}	0.01^{a}	0.02^{a}	0.11^{a}	13.05^{a}	0.05^{b}	0.30^{b}	0.10	3.88	0.66^{b}	36.57	1.16^{b}	36.83^{b}	0.59^{b}	1.05^{b}	0.23^{b}	0.00	0.55^{b}	0.00	0.45^{b}
FAD (*n* = 13)		0.00	0.03^{b}	0.03^{b}	0.29^{b}	1.21^{b}	18.90^{b}	0.03^{a}	0.16^{a}	0.08	4.50	0.21^{a}	35.68	0.61^{a}	8.49^{a}	0.27^{a}	0.34^{a}	0.12^{a}	0.01	0.04^{a}	0.00	0.03^{a}
p values [3]		0.433	0.029	0.001	0.000	0.000	0.001	0.001	0.008	0.079	0.594	0.000	0.173	0.000	0.000	0.000	0.000	0.000	0.388	0.000	0.477	0.000

Abbreviations: SCP, blends of AO from seed oils, cocoa butter and palm oil; SP, blends of AO from seed and palm oils; O, AO from olive pomace oil and blends of AO from olive pomace and olive oils; BS, blends of AO from seed oils; SU, AO from sunflower oil; SU-SO, blends of AO from sunflower and soybean oils; SO, AO from soybean oil; LFAD, FAD from coconut oil and blends of FAD from coconut and palm kernel oils; PFAD, FAD from palm oil; OFAD, FAD from olive pomace and olive oils. [1] p values were obtained from Kruskal-Wallis U test. Stepwise Multiple Comparisons for independent samples were used to compare medians between botanical origin groups within acid oils (AO, *n* = 79). AO groups bearing different letters (a-d) were significantly different ($p \leq 0.05$). [2] p values were obtained from Kruskal-Wallis U test. Stepwise Multiple Comparisons for independent samples were used to compare medians between botanical origin groups within fatty acid distillates (FAD, *n* = 13). FAD groups bearing different letters (a-c) were significantly different ($p \leq 0.05$). [3] p values were obtained from Mann Whitney U test for independent samples to compare medians between AO and FAD (AO, *n* = 79; FAD, *n* = 13). Groups bearing different letters (a-b) were significantly different ($p \leq 0.05$).

Table S2. Mean, standard deviation, median, minimum and maximum values for the different sums of fatty acids identified and quantified by peak area normalization (%) according to the botanical origin of the acid oils (AO).

		SFA (%)	*trans*-C18:1	*cis*-MUFA (%)	n-6 PUFA (%)	n-3 PUFA (%)	UFA/SFA ratio
SCP	Mean ± SD	46.4 ± 3.04	0.6 ± 0.32	39.2 ± 1.59	12.9 ± 1.40	0.9 ± 0.16	2.7 ± 1.72
	Median	47.2^{d}	0.5^{ab}	38.8^{c}	12.7^{b}	0.9^{a}	2.7
	Min	39.1	0.2	37.6	11.2	0.6	0.9
	Max	49.1	1.4	43.3	15.5	1.0	4.5
SP	Mean ± SD	29.8 ± 6.36	0.6 ± 0.61	43.8 ± 3.27	24 ± 4.79	1.9 ± 1.02	2.5 ± 1.31
	Median	27.3^{c}	0.3^{ab}	45.5^{c}	24.9^{c}	1.8^{b}	2.4
	Min	22.8	0.1	39.1	18.4	0.7	1.0
	Max	39.2	1.4	46.7	30.8	3.3	4.1
O	Mean ± SD	18.8 ± 0.66	1.6 ± 0.89	67.3 ± 1.13	11.3 ± 1.00	1 ± 0.09	4.3 ± 1.31
	Median	18.6^{b}	1.7^{c}	67.4^{d}	10.9^{a}	1.1^{ab}	4.7
	Min	18.1	0.3	65.5	10.2	0.8	0.9
	Max	20.2	2.9	69.5	14.2	1.2	5.5
BS	Mean ± SD	17.7 ± 2.68	0.9 ± 0.54	39.3 ± 10.36	41.1 ± 11.83	1 ± 0.42	3.3 ± 1.88
	Median	18.2^{ab}	0.7^{abc}	34.9^{bc}	45.8^{d}	0.9^{ab}	4.1
	Min	14.2	0.2	30.1	18.9	0.4	1.1
	Max	22.5	1.6	59.5	53.2	1.6	6.1
SU	Mean ± SD	17.2 ± 1.23	1.2 ± 0.98	35 ± 6.98	45 ± 7.12	1.6 ± 1.05	3.9 ± 1.61
	Median	17.1^{a}	1^{bc}	33.4^{b}	46.2^{d}	1.3^{b}	4.4
	Min	15.0	0.1	26.7	24.7	0.4	0.2
	Max	19.2	4.5	55.8	54.5	3.7	5.5
SU-SO	Mean ± SD	17.9 ± 1.39	0.4 ± 0.36	27.7 ± 5.45	51.9 ± 5.27	2.2 ± 1.31	3 ± 2.27
	Median	17.6^{ab}	0.4^{a}	23.4^{a}	52.7^{e}	2.0^{b}	3.6
	Min	16.0	0.1	22.7	41.6	0.2	0.1
	Max	20.9	1.1	37.6	59.2	4.0	5.9
SO	Mean ± SD	18.1 ± 2.56	0.6 ± 0.73	26.7 ± 5.21	51.8 ± 0.12	2.8 ± 3.51	5 ± 0.00
	Median	18.1^{ab}	0.6^{ab}	26.7^{a}	51.8^{e}	2.8^{ab}	5.00
	Min	16.3	0.0	23.1	51.7	0.4	5.00
	Max	19.9	1.1	30.4	51.9	5.3	5.00
p values [1]		<0.001	0.001	<0.001	<0.001	0.021	0.057

Abbreviations: SCP, blends of AO from seed oils, cocoa butter and palm oil; SP, blends of AO from seed and palm oils; O, AO from olive pomace oil and blends of AO from olive pomace and olive oils; BS, blends of AO from seed oils; SU, AO from sunflower oil; SU-SO, blends of AO from sunflower and soybean oils; SO, AO from soybean oil; SFA, saturated fatty acids; cis-MUFA, cis-monounsaturated fatty acids; cis-PUFA, cis-polyunsaturated fatty acids; UFA/SFA ratio, unsaturated/saturated ratio. [1] p values were obtained from Kruskal-Wallis U test. Stepwise Multiple Comparisons for independent samples were used to compare medians between botanical origin groups within acid oils (AO, n = 79). AO groups bearing different letters (a-d) were significantly different ($p \leq 0.05$).

Table S3. Mean, standard deviation, median, minimum and maximum values for the different sums of fatty acids identified and quantified by peak area normalization (%) according to the botanical origin of the fatty acid distillates (FAD).

		SFA (%)	*trans*-C18:1	*cis*-MUFA (%)	n-6 PUFA (%)	n-3 PUFA (%)	UFA/SFA ratio
LFAD	Mean ± SD	86.3 ± 1.4	0.2 ± 0.16	10.8 ± 1.57	2.6 ± 0.24	0.1 ± 0.02	3.9 ± 3.40
	Median	87.1[c]	0.1	9.9[a]	2.5[a]	0.1[a]	4.7
	Min	84.3	0.1	9.6	2.3	0.0	0.2
	Max	87.6	0.5	13.2	2.9	0.1	6.8
PFAD	Mean ± SD	53 ± 1.78	0.2 ± 0.07	37.6 ± 1.84	8.9 ± 0.31	0.3 ± 0.02	4.1 ± 0.55
	Median	53.4[b]	0.2	37[b]	8.9[b]	0.3[b]	4.2
	Min	50.0	0.2	35.9	8.5	0.3	3.4
	Max	55.1	0.3	41.0	9.3	0.4	4.6
OFAD	Mean ± SD	14.3 ± 2.14	0.2 ± 0.01	74.3 ± 3.44	10.2 ± 0.89	0.9 ± 0.39	2.6 ± 2.42
	Median	14.3[a]	0.2	74.3[c]	10.2[c]	0.9[c]	2.6
	Min	12.8	0.2	71.9	9.6	0.7	0.9
	Max	15.8	0.2	76.8	10.9	1.2	4.3
p values [1]		0.006	0.493	0.006	0.006	0.006	0.607

Abbreviations: LFAD, FAD from coconut oil and blends of FAD from coconut and palm kernel oils; PFAD, FAD from palm oil; OFAD, FAD from olive pomace and olive oils; SFA, saturated fatty acids; cis-MUFA, cis-monounsaturated fatty acids; cis-PUFA, cis-polyunsaturated fatty acids. [1] p values were obtained from Kruskal-Wallis U test. Stepwise Multiple Comparisons for independent samples were used to compare medians between botanical origin groups within fatty acid distillates (FAD, $n = 13$). AO groups bearing different letters (a-c) were significantly different ($p \leq 0.05$).
